# Circular RNA hsa_circ_0003288 induces EMT and invasion by regulating hsa_circ_0003288/miR-145/PD-L1 axis in hepatocellular carcinoma

**DOI:** 10.1186/s12935-021-01902-2

**Published:** 2021-04-15

**Authors:** Guili Xu, Peng Zhang, Hansi Liang, Yunhua Xu, Jian Shen, Wansheng Wang, Mingming Li, Jintao Huang, Caifang Ni, Xueguang Zhang, Xiaoli Zhu

**Affiliations:** 1 Department of Interventional Radiology, First Affiliated Hospital of Soochow University, 188 Shizi Street, Suzhou, Jiangsu 215006 China; 2 Jiangsu Institute of Clinical Immunology, The First Affiliated Hospital of Soochow University, Suzhou, Jiangsu 215006 China

**Keywords:** Hepatocellular carcinoma, Hsa, Circ, 0,003,288, MiR-145, PD-L1, EMT

## Abstract

**Background:**

Epithelial-mesenchymal transition (EMT) has been associated with wound healing, tumorigenesis, and metastasis. Circular RNAs (circRNAs) are functional non-coding RNAs involved in multiple human cancers. However, whether and how circRNAs contribute to the EMT in hepatocellular carcinomas (HCC) remains to be deciphered. In this study, we investigated the regulation and function of hsa_circ_0003288 on programmed death-1 ligand 1 (PD-L1) during EMT and HCC invasiveness.

**Methods:**

Hsa_circ_0003288 expression was measured by real-time quantitative reverse transcriptase PCR (qRT-PCR). Luciferase reporter assays, RNA pull-down assay and fluorescence in situ hybridization (FISH) were used to determine the correlation between hsa_circ_0003288 and miR-145 and between miR-145 and PD-L1. Furthermore, ectopic overexpression and siRNA-mediated downregulation of hsa_circ_0003288, transwell assays, and in vivo studies were used to determine the function of hsa_circ_0003288 on the EMT and invasiveness of L02 and HCC cells.

**Results:**

miR-145 directly targeted the PD-L1 3′-untranslated region (UTR) region, and hsa_circ_0003288 acted as a miR-145 sponge to regulate PD-L1 expression. Overexpression of hsa_circ_0003288 increased PD-L1 levels and promoted EMT, migration, and invasiveness of L02 cells. These observations were reversed after knockdown of hsa_circ_0003288 in HepG2 and Huh7 cells. Overexpression of PD-L1 rescued EMT, migration, and invasiveness of HepG2 and Huh7 cells after knockdown of hsa_circ_0003288. Furthermore, hsa_circ_0003288 knockdown reduced EMT in in vivo studies. Hsa_circ_0003288/PD-L1 axis was found to mediate the metastatic phenotypes via the PI3K/Akt pathway in HCC. Additionally, expression levels of hsa_circ_0003288 were increased and positively correlated with PD-L1 expression in HCC tissues.

**Conclusion:**

Our findings demonstrated that hsa_circ_0003288 promoted EMT and invasion of HCC via the hsa_circ_0003288/miR-145/PD-L1 axis through the PI3K/Akt pathway. Targeting hsa_circ_0003288 may be a therapeutic strategy for the treatment of HCC.

## Background

Hepatocellular carcinoma (HCC) is the second leading cause of cancer-related mortality worldwide, especially in China [1]. Rapid invasion, metastasis, and recurrence are some of the characteristics of HCC that makes it difficult to treat. EMT plays an important role in tumor invasion and metastasis [2]. Understanding the possible molecular mechanisms underlying EMT in HCC may help identify promising therapeutic targets for HCC.

Epithelial-mesenchymal transition (EMT) plays an important role in cancer progression. During the EMT process, epithelial cancer cells lose their epithelial properties, such as cell polarity and intercellular adhesion, and gain mesenchymal properties, such as migration and invasion ability. Importantly, EMT has been associated with drug resistance of targeted therapies [3, 4]. EMT is important for HCC metastasis and has been closely associated with patient survival. Previous studies have suggested that sorafenib resistance may be due to the EMT process [5]. The development of sorafenib resistance in Huh7 cells may involve the PI3K/Akt signaling pathway. The PI3K/Akt signaling pathway works in parallel with the Raf/Ras/MAPK signaling pathway and is targeted by sorafenib [6]. EMT is coordinated by multiple EMT-induced transcription factors (EMT-TFs). These transcription factors have been shown to be involved in stemness, immune escape, and drug resistance [7], such as ZEB1/2, SNAI1/2, and TWIST1/2 [8].

Circular RNAs (CircRNA) are endogenous, abundant, and stable novel non-coding RNAs (ncRNAs) that are present in a variety of cell types. They have been closely associated with several diseases and cancers [9, 10]. Studies have demonstrated the unique biogenetic mechanisms and functions of circRNAs. Compared to circular microRNA (miRNA) in the cytoplasm which acts as sponges [11], nuclear-localized circRNAs act as transcriptional activators [12]. circRNAs act as natural miRNA sponges by competitively binding with miRNA response elements to inhibit their function. circ-CSPP1 has been shown to reduce the inhibitory effect of miR-1236-3p on ZEB1 to promote EMT and ovarian cancer development. Circ-CSPP1 accomplishes this by acting as a miR-1236-3p sponge [13]. Hsa_circ_0020397 has been shown to regulate cell viability and invasion of colorectal cancer (CRC) cells by sponging miR-138 to promote TERT and PD-L1 expression [14]. Numerous studies have indicated that circRNAs are differentially expressed in HCC and play a key role in several biological processes of HCC. This suggests that circRNAs may be valuable diagnostic markers and/or therapeutic targets for HCC [15–19]. A previous study demonstrated that HCC patients with higher expression levels of hsa_circ_0003288 were associated with poor prognosis [20].

In this study, we investigated the role and mechanism of hsa_circ_0003288 on EMT, migration, and invasion of HCC, as well as the association between hsa_circ_0003288 and PD-L1 in HCC. Our results demonstrated that higher expression of hsa_circ_0003288 induced PD-L1 expression by acting as a sponge for miR-145. Furthermore, hsa_circ_0003288 promoted EMT, migration, and invasion by regulating PD-L1 in HCC. These findings provide strong evidence that hsa_circ_0003288 may be a valuable therapeutic target for the treatment of HCC.

## Materials and methods

### Cell culture

Human HCC cell lines, HepG2, Huh7, SMMC-7721, and Bel-7402 and human normal hepatic cell line, L02, were purchased from the cell bank of the Chinese Academy of Sciences. Cells were cultured in DMEM (high glucose) medium (Gibco BRL, Grand Island, NY) with 100 U/mL penicillin, 100 μg/mL streptomycin, and 10% heat-inactivated FBS (fetal bovine serum) (Invitrogen, Carlsbad, CA, USA) in a cell culture incubator with 5% CO_2_ at 37 °C.

### Tissue samples

Forty matched HCC and adjacent non-tumor liver tissues were obtained from the First Affiliated Hospital of Soochow University after informed consent from patients and Ethics Committee approval was obtained. Of the 40 patients, 18 patients were females and 22 were males. The average age was 58.26 years old. HCC tissues were evaluated by pathological examination. HCC patients did not undergo chemotherapy or radiotherapy prior to tissue sampling. Tissue samples were snap-frozen in liquid nitrogen until required.

### Western blot assays

Total proteins were extracted from tissue samples using RIPA buffer (Cell Signaling Technology) supplemented with protease inhibitors (Sigma Aldrich, St. Louis, MO, USA). Total proteins were then separated on a 10% sodium dodecyl sulfate–polyacrylamide gel electrophoresis (SDS-PAGE) and then transferred to a nitrocellulose membrane (Millipore, Bedford, MA, USA). The membranes were blocked using 5% BSA in TBST buffer and incubated with the following primary antibodies: mouse anti-E-cadherin (610181A) and anti-N-cadherin (610,920, BD Biosciences, San Jose, CA, USA), rabbit anti-PD-L1 (13684S), anti-AKT (9272S), and anti–p-AKT (4060S) (Cell Signaling Technology, Houston, TX, USA), mouse anti-β-actin (sc-47778) and anti-mouse (sc-2005). Membranes were then washed and incubated with the appropriate secondary antibody conjugated with HRP: anti-mouse (sc-2005) and anti–rabbit (sc-2357, Santa Cruz Biotechnology). Specific protein bands were visualized using the ECL reagent (GE Healthcare Life Sciences, Piscataway, NJ, USA). Protein expression levels were normalized against β-actin levels. All experiments were performed in triplicate.

### Real-time quantitative reverse transcriptase PCR (qRT-PCR)

Total RNA was isolated from tissue samples and cells using the HP Total RNA Kit (Omega Biotech, Stamford, CT, USA) based on the manufacturer’s instructions. Quantitation of total RNA was performed using the NanoDrop (Thermo Fisher Scientific, Waltham, MA, USA). cDNA was synthesized from total RNA using the M-MLV First Strand kit (Life Technologies, Gaithersburg, MD, USA). Platinum®SYBR®Green qPCR supermix-UDG with ROX (Invitrogen) was used for PCR amplification and was performed on the ABI PRISM 7500 Sequence Detection System (Applied Biosystems). PCR primer sequences are listed below:circ_0003288-F:5′-TGCATCTTTCTTCAACATGTCCT, circ_0003288-R:5′-ATGCGTGCAAGAACCTTTCA;

miR-145-F: GTCCAGTTTTCCCAGGA, miR-145-R: GAACATGTCTGCGTATCTC; PD-L1-F: 5′- GCTGCACTAATTGTCTATTGGGA, PD-L1-R: 5′- AATTCGCTTGTAGTCGGCACC; β-actin-F:5′-GGCGGCACCACCATGTACCCT, β-actin-R:5′- AGGGGCCGGACTCGTCATACT.

PCR cycling conditions were as follows: 50 °C for 2 min, 95 °C for 10 min, followed by 40 cycles consisting of 95 °C for 15 s and 60 °C for 1 min. U6 expression levels were used to normalize miRNA expression levels. β-actin was used as the endogenous control for mRNA and circRNA expression levels. Relative RNA expression was calculated based on the 2^−ΔΔCt^ method.

### Luciferase reporter assays

PD-L1 3′-UTR and hsa_circ_0003288 sequences containing the miR-145 binding sites were cloned into the psiCHECK2 dual-luciferase vector (Promega, Madison, WI, USA). DNA fragments of PD-L1-3′-UTR, PD-L1-3′-UTR-mutant, hsa_circ_0003288, and hsa_circ_0003288-mutant were directly synthesized (GENEWIZ Inc., Suzhou, China), and then subcloned into the psiCHECK-2 vector. Afterward, the constructs were co-transfected with miR-145 mimics or the corresponding negative controls (miR-NC) into HepG2 and Huh-7 cells. Transient transfections of the miR-145 inhibitor and its corresponding negative control (anti-miR-NC) were performed using Lipofectamine 2000 (Invitrogen, Carlsbad, California, USA). After 48 h post-transfection, cells were harvested and luciferase activity was measured using the Dual-Luciferase Reporter Assay Kit (Promega). Data were presented as relative Renilla luciferase activity normalized against firefly luciferase activity.

### RNA pull-down assay

A biotinlabeled hsa_circ_0003288 probe was synthesized directly by RiboBio Co., Ltd. The probe sequence was Bio-5′-AAGAACCTTTCAGTTGAAAG (lnc1102984, RiboBio, Guangzhou, China). Hsa_circ_0003288 was transfected with Lipofectamine 2000 reagent (Invitrogen thermo, USA) into HepG2 cells. Then cells were harvested and lysed in lysis buffer. After centrifugation, 50 μL supernatant were drained as the input, and the remaining were incubated with streptavidin Dynabeads (M-280, Invitrogen) conjugated with biotin-labeled hsa_circ_0003288 probe overnight at 4 °C. Finally, the total RNA was isolated and miR-145 expression was analyzed by qRT-PCR assay.

### Fluorescence in situ hybridization (FISH)

In situ hybridization was performed with a Fluorescent In Situ Hybridization (FISH) Kit (RiboBio, Guangzhou, China). Cells were washed with PBS buffer solution and fixed in 4% paraformaldehyde for 10 min. And then, the cells were infiltrated with PBS containing 0.5% Triton X-100 at 4 °C for 5 min, washed with PBS three times, each time for 5 min, and prehybridized at 37 °C for 30 min before hybridization. An anti-hsa_circ_0003288, anti-18S, or anti-U6 oligodeoxynucleotide probe was added into the hybridization solution at 37 °C darkness overnight. The cells were incubated with DAPI for10 min. Hsa_circ_0003288-cy3 FISH probes were designed and synthesized directly by RiboBio Co., Ltd. Mouse 18S FISH probes and mouse U6 FISH probes were served as the cytoplasmic and nuclear controls, respectively. Images were captured by a Leica TCS SP8 confocal imaging system (Leica, Germany).

### Generation of L02 cells overexpressing hsa_circ_0003288

L02 cell lines were generated to overexpress hsa_circ_0003288. Briefly, full-length hsa_circ_0003288 was subcloned into the pLCDH-ciR lentiviral expression vector (Geneseed Biotech) to establish the pLCDH-hsa_circ_0003288-copGFP(T2A)Puro construct. We used GENEWIZ inc. to directly synthesize the subcloned sequence of the front circular frame (SA) and back circular frame (SD) of circRNA and full-length of hsa_circ_0003288. The constructs were transiently transfected into cells using Lipofectamine 2000. An empty vector served as the negative control.

### hsa_circ_0003288 knockdown using RNAi

A short interfering RNA (siRNA) against hsa_circ_0003288 (si-hsa_circ_0003288: 5′-CUGAAAGGUUCUUGCACGCTT-3′) was used to knockdown hsa_circ_0003288 expression and was synthesized by GenePharma, Shanghai, China. Scrambled siRNA (5′-GACUUUCCUUCUUGCACGCTT-3′) served as a negative control. HCC cells were transiently transfected with 100 pmol of siRNA using Lipofectamine 2000 reagent (Invitrogen).

### Transwell migration and invasion assays

Invasion and migration assays were performed using transwell plates (353,097, BD Falcon, USA). 24-well transwell chambers with 8 mm pore polycarbonate filters were coated with or without Matrigel for invasion and migration assays, respectively. 5 × 10^4^ cells were cultured in DMEM media with 1% FBS and placed on the upper chamber. DMEM media supplemented with 10% FBS was added to the bottom chamber, which served as the chemoattractant. After 48 h, media was aspirated and cells that adhered to the upper surface of the membrane were gently removed using a cotton swab. Cells that had attached to the lower layer of the membrane were then stained using 1% crystal violet for 30 min at room temperature and then imaged using a light microscope. Results were obtained from three independent experiments.

### In vivo studies

Female BALB/c nude mice (6–8 weeks) were divided into two groups, i.e., the stable hsa_circ_0003288 silencing group and the control group (5 mice per group). The right flank of mice was subcutaneously injected with 2 × 10^5^ HepG2 cells transfected with the control vector or si-hsa_circ_0101145. 7 days post-injection, tumors that formed were measured (volume = 1/2 × length × width^2^) using a vernier caliper every 5 days. After 1 month, mice were euthanized by cervical dislocation, and tumors were excised and used for protein extraction and western blot analysis. All animal experiments were approved by the Ethics Committee of the First Affiliated Hospital of Soochow University.

## Statistical analysis

Differences between the two groups were determined using paired or unpaired t-test (two-tailed). Association between the two groups was determined using Pearson’s correlation test. Results were presented as mean ± SEM and *P* < 0.05 was considered significant. Statistical analyses were performed using GraphPad Prism 5.02 software (GraphPad, San Diego, CA, USA).

## Results

### Hsa_circ_0003288 promotes EMT, migration, and invasion of HCC

**Fig. 1:**
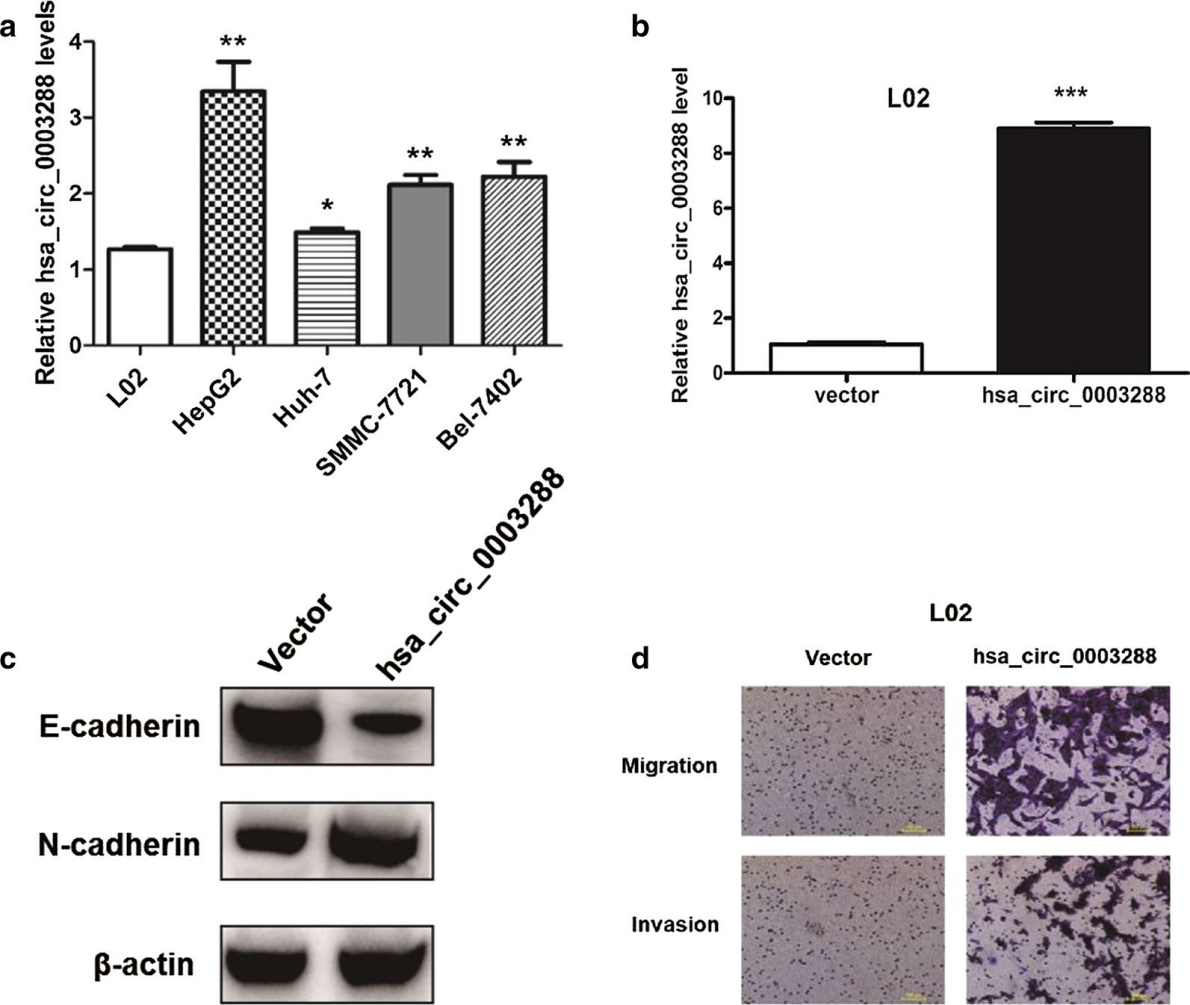
Hsa_circ_0003288 promotes HCC EMT, migration, and invasion. **a** qRT-PCR analysis of hsa_circ_0003288 expression levels in HCC cell lines HepG2, Huh-7, SMMC-7721, Bel-7402, and human normal hepatic cell line L02. Relative expression levels of hsa_circ_0003288 normalized against β-actin levels. **b** Hsa_circ_0003288 expression levels in L02 cells transiently overexpressing hsa_circ_0003288 measured by qRT-PCR. Empty vector was used as the negative control. **c** L02 cells were transfected with hsa_circ_0003288 for 48 h. E-cadherin and N-cadherin protein levels were measured by western blot. β-actin served as the loading control. **d** Transwell assays were performed on L02 cells transiently overexpressing hsa_circ_0003288. Scale bar, 100 μm. *P < 0.05; **P < 0.01; ***P < 0.001

Previous studies have demonstrated that HCC patients with higher expression of hsa_circ_0003288 have a poor prognosis [20]. EMT has been shown to be a key driver of tumor cell invasion and metastasis [21]. To investigate the biologic function of hsa_circ_0003288 in regulating EMT, we first measured hsa_circ_0003288 expression in HCC cell lines by qRT-PCR. Our results demonstrated that hsa_circ_0003288 expression was higher in HCC cell lines compared to normal L02 cells (Fig. 1a). Next, we overexpressed hsa_circ_0003288 in L02 cells (Fig. 1b). Western blot analysis showed that overexpression of hsa_circ_0003288 reduced the epithelial marker E-cadherin expression levels but increased mesenchymal marker N-cadherin expression levels (Fig. 1c). In addition, transwell assays demonstrated that overexpression of hsa_circ_0003288 promoted cell migration and invasion in L02 cells (Fig. 1d). Taken together, these results suggested that hsa_circ_0003288 may be associated with EMT in HCC cells.

### Hsa_circ_0003288 binds directly to miR-145 in HCC cells

**Fig. 2:**
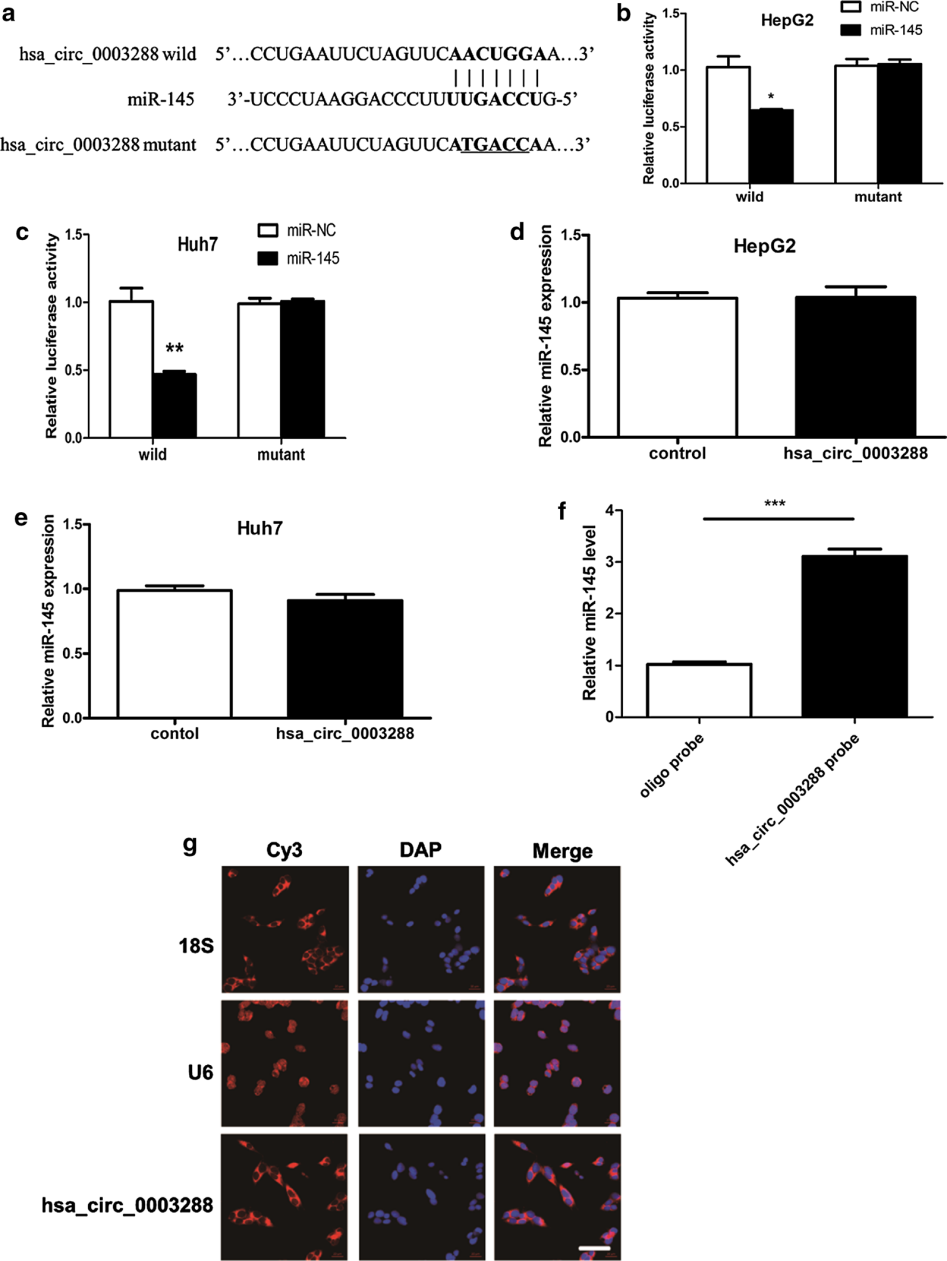
Hsa_circ_0003288 bind directly to miR-145 in HCC cells. **a** Predicted target sequences in hsa_circ_0003288 binding to miR-145. **b**, **c** Wild type or mutated hsa_circ_0003288 transfected into HepG2 or Huh-7 cells with miR-145 or negative control (miR-NC). Relative luciferase activity was determined 48 h post-transfection. U6 was used as the internal control. **d**, **e** Endogenous miR-145 levels in HepG2 and Huh-7 cells after transient overexpression of hsa_circ_0003288. **f** Cell lysates from HepG2 cells were incubated with biotinylated probes against hsa_circ_0003288; miR-145 enrichments was determined by qRT-PCR after pull-down. Results revealed that miR-145 was abundantly pulled down by the hsa_circ_0003288 probe in HepG2 cells. **g** The subcellular distribution of hsa_circ_0003288 was visualized by RNA Fluorescent in situ hybridization (FISH) in HepG2 cells. 18S was the positive control for cytoplasm, and U6 was the positive control for the nucleus. Scale bar: 20 μm. *P < 0.05; **P < 0.01

Endogenous circRNAs have been shown to function as miRNA sponges in various cancers [22]. We investigated the molecular mechanism by which hsa_circ_0003288 mediates EMT in HCC cells. Bioinformatic analysis was used to predict candidate miRNAs, which showed that hsa_circ_0003288 could interact with several miRNAs, such as miR-1179, miR-1200, miR-1233, miR-136, miR-145, miR-1203, and miR-942. To determine whether hsa_circ_0003288 could directly bind to miR-145 in HCC cells, the hsa_circ_0003288 region containing putative miR-145 binding sites (wild type/mutant) (Fig. 2a) were subcloned into the psiCHECK-2 luciferase vector and then transiently co-transfected with miR-145 mimics into HepG2 and Huh7 cells. Our results demonstrated that the psiCHECK2 luciferase reporter construct with the hsa_circ_0003288 wild type had a lower luciferase activity in HepG2 cells. However, luciferase activity did not change significantly in cells transfected with the mutated binding sites (Fig. 2b). Similar results were obtained using Huh7 cells (Fig. 2c). We then transiently overexpressed hsa_circ_0003288 (Fig. 1b) in HepG2 and Huh7 cells. We found that the differences in miR-145 levels were not significant between cells overexpressing hsa_circ_0003288 and the control (Fig. 2d, e). RNA pull-down assays demonstrated that endogenous miR-145 was pulled down by biotinylated probe against hsa_circ_0003288 in HepG2 cells (Fig. 2f). The fluorescence in situ hybridization (FISH) experiments showed that hsa_circ_0003288 was mainly distributed in plasma, suggesting that hsa_circ_0003288 might play a post-transcriptional regulation role (Fig. 2g). Taken together, these suggested the binding of hsa_circ_0003288 with miR-145. Taken together, our findings demonstrated that hsa_circ_0003288 could act as a sponge for miR-145 in HCC cells.

### miR-145 represses PD-L1 expression by directly targeting the 3′-UTR of PD-L1 in HCC cells

**Fig. 3:**
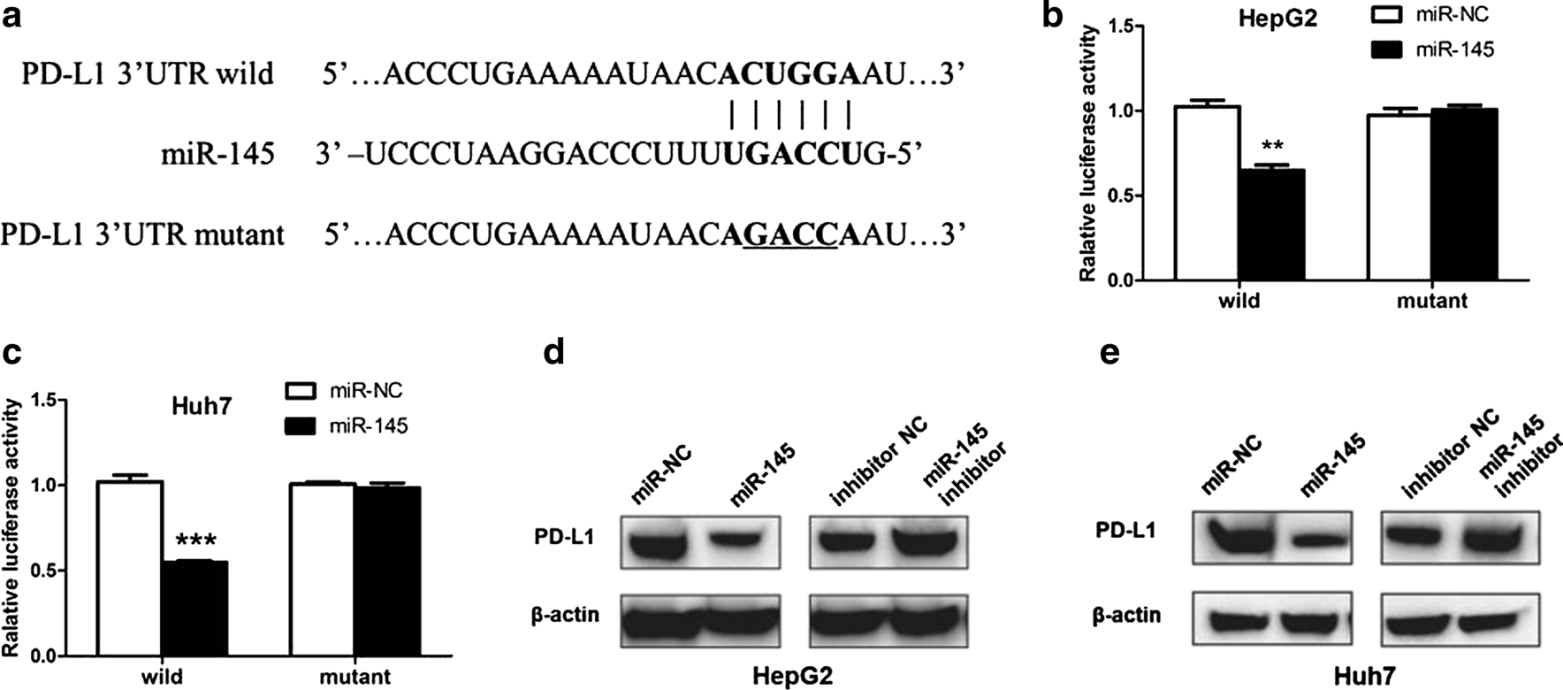
miR-145 inhibits PD-L1 expression by directly targeting the 3′-UTR of PD-L1 in HCC cells. **a** Predicted target sequences of PD-L1 3′-UTR binding to miR-145. **b** and **c** Wild type or mutated PD-L1 3′-UTR transfected into HepG2 or Huh-7 cells with miR-145 or negative control (miR-NC). Relative luciferase activity was determined 48 h post-transfection. **d** and **e** PD-L1 protein levels in HepG2 and Huh-7 cells transfected with miR-145 mimics, miR-NC, miR-145 inhibitor or inhibitor NC. **P < 0.01; ***P < 0.001

Previous studies have shown that PD-L1 plays an important role in EMT transition in lung and head and neck squamous cell carcinomas [23, 24]. Bioinformatics analysis predicted several miRNAs that could potentially target the 3′-UTR of PD-L1 mRNA. These included miR-200a, miR-200c, miR-20a, miR-145, miR-17, miR-519d, and miR-93. miR-145 has been previously demonstrated to be involved in regulating the EMT process in several cancers [25, 26]. Hence, we selected miR-145 for further study. We subcloned PD-L1 mRNA 3′-UTR containing the miR-145 binding site (wild type/mutant) into psiCHECK-2 vector (Fig. 3a), and then transiently co-transfected the reporter construct with miR-145 into HepG2 and Huh7 cells. Our results demonstrated that miR-145 could reduce the luciferase activity of PD-L1 3′-UTR wild type but not the PD-L1 3′-UTR mutant. This was observed both in HepG2 and Huh7 cells (Fig. 3b, c). Furthermore, overexpression of miR-145 using miR-145 mimics decreased PD-L1 expression, while knockdown of miR-145 using miR-145 inhibitor mimics increased PD-L1 levels in HepG2 and Huh7 cells (Fig. 3d, e). Taken together, these findings suggest that miR-145 could suppress PD-L1 expression by directly targeting the 3′-UTR of PD-L1 in HCC cells.

### Hsa_circ_0003288 increases PD-L1 expression by acting as a miR-145 sponge

**Fig. 4:**
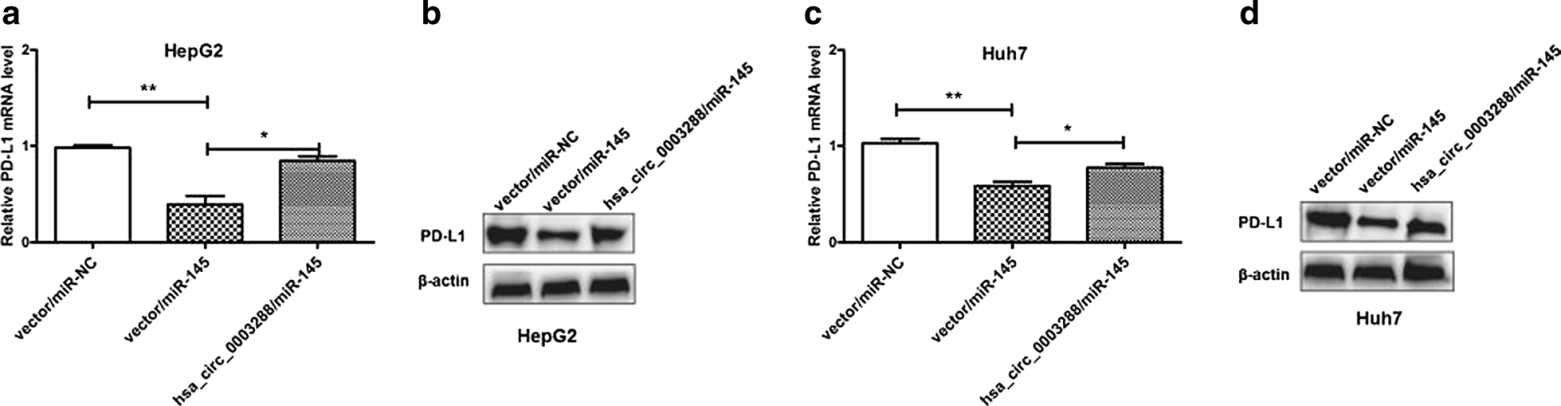
Hsa_circ_0003288 increases PD-L1 expression levels by sponging miR-145. **a** and **c** Hsa_circ_0003288 reverses miR-145 induced inhibition of PD-L1 mRNA expression measured using qRT-PCR in HepG2 and Huh7 cells. **b** and **d** Hsa_circ_0003288 reverses miR-145 induced inhibition of PD-L1 protein expression measured using western blotting in HepG2 and Huh7 cells

To further investigate the role of hsa_circ_0003288, we investigated whether hsa_circ_0003288 could attenuate endogenous miR-145-mediated suppression of PD-L1. We overexpressed hsa_circ_0003288 in HepG2 and Huh7 cells transfected with miR-145 mimics and measured PD-L1 levels. Our results demonstrated that overexpression of miR-145 could reduce PD-L1 expression levels, while overexpression of hsa_circ_0003288 could prevent inhibition of PD-L1 expression in HepG2 and Huh7 cells (Fig. 4a–d). These results demonstrate that hsa_circ_0003288 could reduce the inhibitory effects of miR-145 on PD-L1 expression by functioning as a miR-145 sponge in HCC cells.

### miR-145 inhibits EMT, migration, and invasion of HCC cells

**Fig. 5:**
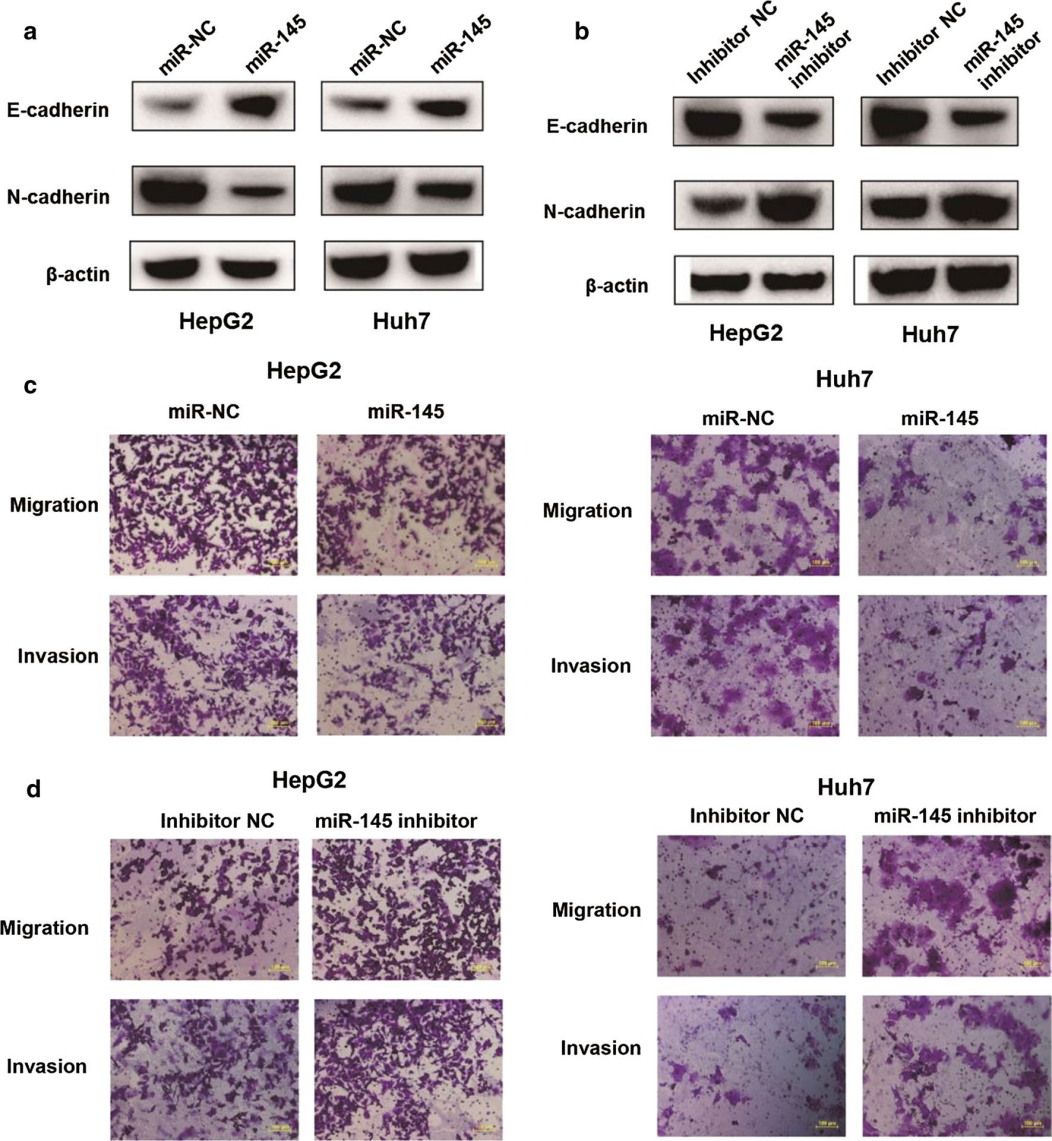
miR-145 inhibits EMT, migration, and invasion of HCC cells. **a** HepG2 and Huh7 cells were transiently transfected with miR-145 mimics or miR-NC. After 48 h post-transfection, western blot analysis was performed to measure levels of E-cadherin and N-cadherin. Expression levels were normalized against β-actin levels. **b** HepG2 and Huh7 cells were transiently transfected with miR-145 inhibitor or inhibitor NC. After 48 h post-transfection, western blot analysis was performed to measure levels of E-cadherin and N-cadherin. Expression levels were normalized against β-actin levels. **c** Migration (through the 8-μM pore) or invasion (through the matrigel-coated membrane) of HepG2 or Huh7 cells transiently transfected with miR-145 mimics or miR-NC. Scale bar, 100 μm. **d** Migration (through the 8-μM pore) or invasion (through the matrigel-coated membrane) of HepG2 or Huh7 cells transiently transfected with miR-145 inhibitor or inhibitor NC. Scale bar, 100 μm

To determine whether miR-145 could regulate the EMT phenotype in HCC cells, we transfected miR-145 mimics or miR-145 inhibitors into HepG2 and Huh7 cells. HepG2 and Huh7 cells transfected with miR-145 mimics had higher expression levels of E-cadherin and lower expression of N-cadherin compared to cells transfected with miR-NC (Fig. 5a). In contrast, knockdown of miR-145 in HepG2 and Huh7 cells had lower expression levels of E-cadherin and higher expression levels of N-cadherin (Fig. 5b). Furthermore, overexpression of miR-145 reduced the migratory and invasive ability of HepG2 and Huh7 cells (Fig. 5c), while knockdown of miR-145 increased their migration and invasion ability (Fig. 5d). Taken together, these findings indicated that miR-145 could suppress EMT, migration, and invasion of HCC cells.

### Hsa_circ_0003288 knockdown inhibits PD-L1 expression and inhibits EMT, migration, and invasion of HCC cells via the PI3K/AKT signaling pathway

**Fig. 6:**
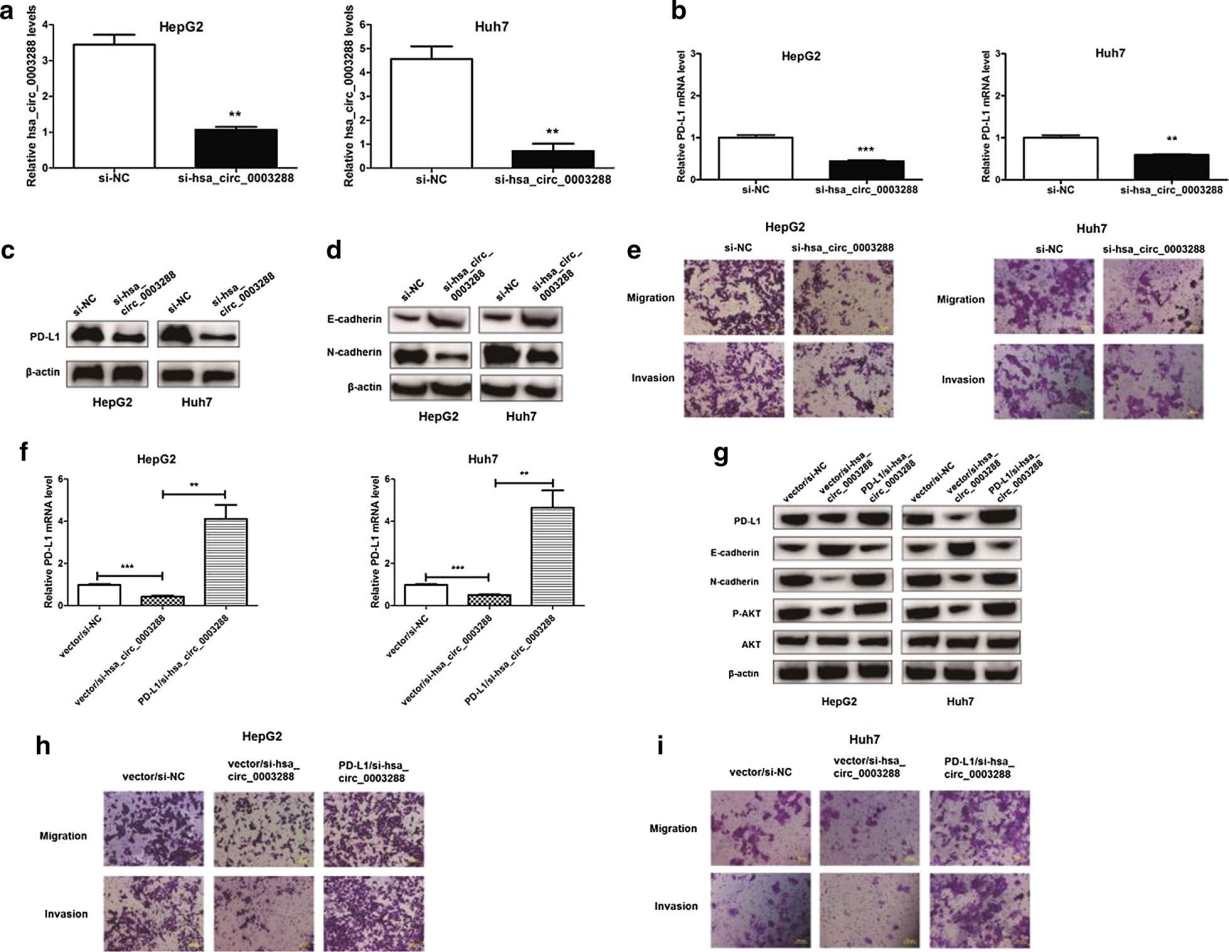
Knockdown of hsa_circ_0003288 inhibits PD-L1 expression and inhibits EMT, migration, and invasion of HCC cells. **a** hsa_circ_0003288 expression levels in HepG2 and Huh7 cells after siRNA transfection. Relative hsa_circ_0003288 expression levels normalized to β-actin levels. **b**, **c** PD-L1 mRNA and protein expression levels in HepG2 and Huh7 cells after siRNA transfection. Relative PD-L1 expression levels normalized to β-actin levels. **d** E-cadherin and N-cadherin protein expression levels in HepG2 and Huh7 cells after siRNA transfection. **e** Transwell migration and invasion assays of HepG2 and Huh7 cells transfected with siRNA or si-NC. Scale bar, 100 μm. **f** qRT-PCR analysis of PD-L1 mRNA expression levels after PD-L1 overexpression in si-hsa_circ_0003288 HepG2 and Huh7 cells. **g** Western blot assay of PD-L1, E-cadherin and N-cadherin, p-AKT and AKT protein levels in HepG2 and Huh7 cells after PD-L1 overexpression in si-hsa_circ_0003288 HepG2 and Huh7 cells. **h** and **i** Transwell migration and invasion assays of si-hsa_circ_0003288 HepG2 and Huh7 cells after PD-L1 overexpression. Scale bar, 100 μm. **P < 0.01; ***P < 0.001

We next investigated whether hsa_circ_0003288 could regulate EMT and invasion in HCC cells. After siRNA knockdown of Hsa_circ_0003288 in HepG2 and Huh7 cells, we observed a significant decrease in PD-L1 mRNA and protein levels (Fig. 6a–c). In addition, hsa_circ_0003288 knockdown increased E-cadherin levels and reduced N-cadherin levels compared to control cells (Fig. 6d). Furthermore, hsa_circ_0003288 knockdown reduced the migratory and invasive ability of HepG2 and Huh7 cells (Fig. 6e). These results suggested that reducing hsa_circ_0003288 levels could decrease PD-L1 expression and inhibit EMT and invasion of HCC cells.

We then overexpressed PD-L1 (Fig. 6f, g) in si-hsa_circ_0003288 HCC cells to determine whether hsa_circ_0003288-induced metastatic phenotypes could be rescued. Western blot assays (Fig. 6g) demonstrated that PD-L1 overexpression led to the recovery of EMT in both HepG2 and Huh7 cells after hsa_circ_0003288 silencing. Furthermore, transwell assays demonstrated that PD-L1 overexpression could restore the migration and invasion ability of both HepG2 (Fig. 6h) and Huh7 (Fig. 6i) cells after hsa_circ_0003288 silencing.

We next investigated what downstream signaling pathway PD-L1 participates in to regulate the metastatic phenotype of HCC. The PI3K/Akt pathway has been shown to play an important role in promoting EMT in multiple cancers [4]. Hence, we focused on the PI3K/Akt signaling pathway. Western blot assays were performed to measure p-AKT levels, which is an indicator of the activation of the PI3K/Akt pathway. P-AKT levels were measured in si-hsa_circ_0003288, PD-L1/si-hsa_circ_0003288, and in control HCC cells. We observed that p-AKT and PD-L1 levels were reduced in si-hsa_circ_0003288 HCC cells compared to control cells (Fig. 6g). Overexpression of PD-L1 was found to increase p-AKT levels in PD-L1/si-hsa_circ_0003288 HCC cells compared to si-hsa_circ_0003288 HCC cells (Fig. 6g).

### Hsa_circ_0003288 expression levels are positively correlated with PD-L1 expression in HCC tissues

**Fig.7:**
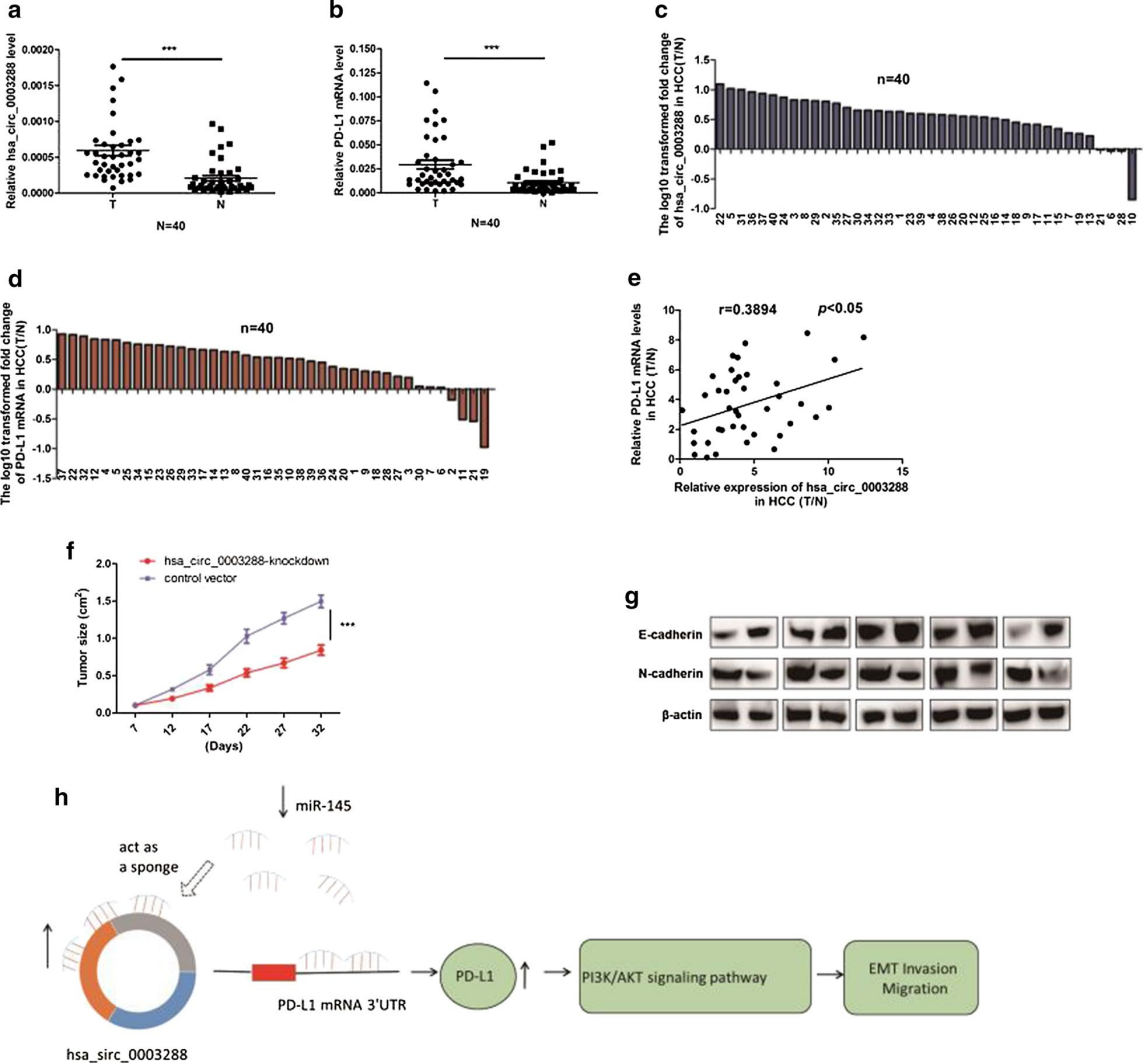
Hsa_circ_0003288 expression levels are positively correlated with PD-L1 expression levels in HCC tissues. **a** and** b** qRT-PCR analysis of hsa_circ_0003288 and PD-L1 mRNA levels in 40 human HCC tissues and paired non-cancerous liver tissues. T, HCC tissues; N, paired non-cancerous liver tissues. **c** and **d** Relative mRNA expression levels of hsa_circ_0003288 and PD-L1 in 40 paired HCC tissues. Y-axis represents log10 transformed fold change of T/N expression ratios of hsa_circ_0003288 and PD-L1 mRNA levels. The ratio for each tissue sample is shown below the x-axis. **e** Correlation between hsa_circ_0003288 expression levels and PD-L1 mRNA levels in 40 paired HCC tissues. X and Y axes represent the T/N expression ratios of hsa_circ_0003288 and PD-L1 mRNA levels, respectively. **f** Tumor volume curve in mice were measured every 5 days in the hsa_circ_0003288-knockdown and control vector groups. **g** E-cadherin (epithelial marker) and N-cadherin expression levels (mesenchymal marker) measure by western blotting. **h** A proposed mechanistic model in which hsa_circ_0003288 functions as a miR-145 sponge and regulates the EMT and invasion via PI3K/Akt pathway in HCC cells. ***P < 0.001

To investigate the relationship between hsa_circ_0003288 and PD-L1, we measured the levels of hsa_circ_0003288 and PD-L1 in 40 HCC and paired non-cancerous tissues using qRT-PCR. HCC tissues were found to have significantly higher hsa_circ_0003288 and PD-L1 mRNA expression levels when compared to paired non-cancerous liver tissues (Fig. 7a, b). Furthermore, the ratio of hsa_circ_0003288 levels (T/N) was positively correlated with PD-L1 mRNA expression levels (T/N) in HCC tissues (Fig. 7e). Importantly, of the 36 HCC tissues with high hsa_circ_0003288 levels, 30 tumors (76.7%) showed higher PD-L1 mRNA levels (Fig. 7c, d). Collectively, these findings suggest that hsa_circ_0003288 plays a key role in regulating PD-L1 expression in HCC.

### Knockdown of hsa_circ_0003288 inhibits in vivo tumor growth and EMT

We next investigated the role of hsa_circ_0003288 in EMT of HCC using mouse models. BALB/c nude mice were subcutaneously injected with HepG2 cells with either stable hsa_circ_0003288-knockdown or control vector. We observed that tumor size was lower in the hsa_circ_0003288-knockdown group compared to the control vector group (Fig. 7f). Western blot assays demonstrated that knockdown of hsa_circ_0101145 increased E-cadherin expression but reduced N-cadherin expression in tumor tissues of mice (Fig. 7g). Our in vivo studies demonstrated that knockdown of hsa_circ_0003288 reduced HCC EMT.

## Discussion

Recent studies have shown that circRNAs are differentially expressed between HCC and normal liver tissues. They play key roles in numerous biological processes, suggesting that circRNAs may be valuable diagnostic or therapeutic targets for HCC [15–19]. However, the underlying mechanism of circRNAs in the development and progression of HCC is yet to be deciphered. In the present study, we demonstrated that hsa_circ_0003288 expression levels were increased in HCC and functioned as a ceRNA to promote EMT and invasion by regulating PD-L1 expression.

CircRNAs are abnormally expressed in several types of cancers, such as lung, liver, and colorectal carcinomas [25–27]. We found that PD-L1 mRNA levels were upregulated in HCC tissues. Our results were consistent with those published by Tao et al. Furthermore, we demonstrated that hsa_circ_0003288 expression levels were significantly increased in HCC cells and tissues and found that increased hsa_circ_0003288 levels were positively correlated with PD-L1 expression levels in HCC tissues. Although circRNA levels are usually expressed at low levels, they play an important role in tumorigenesis and metastasis and may function as oncogenes or tumor suppressors [29–31]. circRNA MTO1 has been shown to inhibit the progression of HCC [28], while circRNA cTFRC has been shown to promote bladder carcinoma progression by acting as a sponge for miR-107 [32]. A previous study demonstrated that HCC patients with high expression levels of hsa_circ_0003288 had poor prognosis [20]. Furthermore, numerous circRNAs have been shown to be regulated during the EMT process in human breast cancer [28]. This suggests that hsa_circ_0003288 maybe have an important role during EMT and invasion of HCC. We demonstrated that L02 cells that transiently overexpressed hsa_circ_0003288 had reduced expression levels of E-cadherin and higher N-cadherin expression levels compared to the control cells. Transwell assays also demonstrated that overexpression of hsa_circ_0003288 increased the migration and invasive ability of L02 cells.

The underlying mechanism of circRNAs in tumorigenesis and progression have not been fully elucidated. Numerous publications have demonstrated that circRNAs act as miRNA sponges and play a role in the cirRNA-miRNA-mRNA axis [26, 29]. circHIPK3 has been reported to be a tumor-related molecule in HCC, and functions as a sponge for miR-124 to control AQP3 expression [17]. hsa_circ_0020397 has been shown to increase the expression of miR-138 targets, TERT and PD-L1, to regulate the growth of colorectal cancer cells [14], while hsa_circ_0008305 (circPTK2) has been shown to function as a sponge for miR-429/miR-200b-3p that directly targets the TIF1 3′-UTR region in non-small cell lung cancer [25]. In the present study, bioinformatics analysis indicated putative binding of hsa_circ_0003288 and miR-145. Dual-luciferase reporter assays confirmed that miR-145 was able to bind to hsa_circ_0003288. And we demonstrated that miR-145 was abundantly pulled down by the hsa_circ_0003288 probe in HepG2 cells. Furhermore, we showed that hsa_circ_0003288 is mainly distributed in the cytoplasm. Additionally, our results demonstrated that miR-145 levels were unchanged after HepG2 and Huh-7 cells were transiently transfected with pLCDH-circPTK2-copGFP(T2A)Puro lentiviral expression vector. This is in agreement with previous studies that suggested circRNAs play an important role in biological and pathological processes by acting as microRNA (miRNA) sponges but not affecting miRNA expression [30]. MiRNAs are small non-coding RNAs that regulate gene expression and are involved in a variety of human cancers. In addition, miRNAs have been demonstrated to be good biomarkers for a variety of cancers [35]. We found that PD-L1 expression levels were down-regulated after transient transfection with miR-145 mimics in HepG2 and Huh7 cells. Furthermore, knockdown of miR-145 by transient transfection with miR-145 inhibitor mimics increased PD-L1 expression levels. Dual-luciferase reporter assays further demonstrated that miR-145 inhibited PD-L1 expression by binding to its 3′-UTR region. The inhibition of PD-L1 expression by miR-145 could be rescued by overexpressing hsa_circ_0003288 in HepG2 and Huh7 cells.

Different molecular mechanisms are involved in the occurrence and development of EMT in human cancer. These include the regulation of non-coding RNAs, EMT transcription factors (EMT-TFs), alterative splicing factors, epigenetic modification, and post-translational regulation [31, 32]. LncRNA PTAR has been shown to regulate ZEB1 expression by competitive binding of miR-101-3p to promote EMT, invasion, and metastasis in ovarian cancers [33]. In this study, miR-145 was found to reduce HCC invasion by inhibiting EMT. Our findings are inconsistent with previous studies where miR-145 was found to promote EMT by regulating Fibroblast Growth Factor 10 (FGF10) [34]. However, our findings were consistent with the results published by Chen et al. where miR-145 was found to inhibit the invasion and metastasis of HCC cells [35]. This is not surprising since miRNAs play different roles depending on the cellular environment and specific targets or downstream effectors [25].

Higher expression levels of PD-L1 has been found in a variety of human cancers and has been closely associated with poor patient prognosis. Programmed death 1 (PD-1) and PD-L1 inhibitors have produced promising clinical results. Compared to paired non-cancerous liver tissues, we observed HCC tissues had significantly higher mRNA expression levels of PD-L1. Interestingly, the PD-L1 mRNA levels were positively correlated with hsa_circ_0003288 expression levels in HCC tissue samples. Previous studies have reported a strong correlation between PD-L1 expression and EMT status in a variety of human cancers [37]. Furthermore, studies have demonstrated that PD-L1 signaling plays an important role in maintaining the EMT status of renal cell carcinomas and breast cancer [38, 39]. We found that knockdown of hsa_circ_0003288 expression reduced PD-L1 mRNA and protein levels in HCC cells. Furthermore, knockdown of hsa_circ_0003288 increased E-cadherin expression levels and reduced N-cadherin levels compared to control cells. Most importantly, knockdown of hsa_circ_0003288 significantly reduced the migration and invasion ability of HCC cells. EMT, migration, and invasion of HepG2 and Huh7 cells were restored after overexpression of PD-L1 following hsa_circ_0003288 silencing. Using in vivo mouse models, we observed that knocking down hsa_circ_0003288 expression in HCC reduced tumor growth and EMT. The PI3K/AKT signaling pathway has been shown to be closely associated with EMT and tumor progression [4]. We demonstrated that p-AKT levels were reduced in si-hsa_circ_0003288 HCC cells, while p-AKT levels increased after PD-L1 was overexpressed in si-hsa_circ_0003288 HCC cells. Taken together, our findings suggest that hsa_circ_0003288 knockdown could decrease PD-L1 expression and inhibit EMT and invasion via the PI3K/AKT signaling pathway in HCC cells.

## Conclusion

In summary, we identified the oncogenic role of hsa_circ_0003288 and its mechanism by which it regulates EMT and invasion of HCC. We found that hsa_circ_0003288 expression levels were significantly increased and positively correlated with PD-L1 expression levels in HCC. hsa_circ_0003288 promoted EMT and invasion of HCC by acting as a miR-145 sponge and upregulating PD-L1 expression levels via the PI3K/AKT signaling pathway. This suggests its oncogenic role in HCC development. Targeting hsa_circ_0003288 may provide a therapeutic strategy for the treatment of HCC.

## Limitations of the study

Due to the limited availability of tumor samples, only PD-L1 mRNA and not protein expression levels were measured. Additional studies using a larger number of patient samples are required to validate our findings.

## Data Availability

The data sets used and/or analyzed during the current study are available from the corresponding author on reasonable request.

## Abbreviations

EMT: Epithelial-mesenchymal transition
circRNAs: Circular RNAs
HCC: Hepatocellular carcinoma
PD-L1: Programmed death-1 ligand 1
qRT-PCR: Real-time quantitative reverse transcriptase PCR
UTR: Untranslated region
EMT-TFs: EMT-induced transcription factors
ncRNAs: Non-coding RNAs
miRNA: MicroRNA
CRC: Colorectal cancer
SDS-PAGE: Sodium dodecyl sulfate–polyacrylamide electrophoresis
siRNA: Short interfering RNA
FGF10: Fibroblast Growth Factor 10
PD-1: Programmed death 1
